# INTEREST GROUP SESSION—SLEEP, CIRCADIAN RHYTHMS AND AGING INTEREST GROUP: SLEEP, PHYSICAL ACTIVITY, AND BRAIN AGING

**DOI:** 10.1093/geroni/igz038.1495

**Published:** 2019-11-08

**Authors:** Christopher N Kaufmann, Katie L Stone

**Affiliations:** 1 Division of Geriatrics and Gerontology, Department of Medicine, University of California San Diego, La Jolla, California, United States; 2 California Pacific Medical Center Research Institute, San Francisco, California, United States

## Abstract

As the US population ages, the prevalence of Alzheimer’s Disease (AD) and related dementias is expected to increase, making dementia prevention efforts a major public health priority. Impaired sleep and circadian rhythms, along with other lifestyle factors, have emerged as important modifiable disease risk factors—recent studies demonstrate the importance of sleep in preventing the development of key biomarkers for AD/dementia pathology. In this symposium, we will highlight findings on the associations of sleep, circadian rhythm disruptions, and daytime activity patterns on development of cognitive decline and dementia, exploring not only the mechanisms driving these associations, but the potential impact of sleep and lifestyle interventions in promoting healthy brain aging. The symposium consists of four presentations which use data from large national cohort studies. First, we will present analyses on patterns of 24-hour (circadian) activity rhythms (e.g., usual time of day for peak activity, regularity of circadian patterns) and incident dementia risk. The second presentation will present findings pertaining to understanding the link between sleep disturbance and inflammation (a substantial contributor to cognitive aging). The third will examine whether detailed daytime activity patterns associate with imaging-based brain volumes, independent of sleep disturbance. The final presentation will explore whether initiation of sleep disorder treatments may have the potential to change trajectories of cognitive performance as individuals age. Overall, the symposium will highlight the importance of sleep and activity patterns to brain health and stimulate discussion about improving sleep and circadian disruption as a target for dementia prevention efforts.

